# Spontaneous cerebral amyloid angiopathy‐related intracerebral hemorrhages in aged capuchin monkey

**DOI:** 10.1002/alz70855_106235

**Published:** 2025-12-24

**Authors:** Roberta Rodriguez, Maria Da Graça Morais Martin, Sonia Maria Dozzi Brucki, Maria Concepción Gracía Otaduy, Leonel Tadao Takada, Lea T. Grinberg, Jessica Mendes de Souza, Claudia Kimie Suemoto, Claudia da Costa Leite, Carlos Tomaz, Maria Clotilde Henriques Tavares, Ricardo Nitrini

**Affiliations:** ^1^ Cognitive and Behavioral Neurology Unit, Hospital das Clinicas HCFMUSP, Faculdade de Medicina, Universidade de Sao Paulo, Sao Paulo, Sao Paulo, Brazil; ^2^ LIM44, Hospital das Clinicas HCFMUSP, Faculdade de Medicina, Universidade de Sao Paulo, Sao Paulo, Sao Paulo, Brazil; ^3^ Biobank for Aging Studies of the University of São Paulo, Sao Paulo, Sao Paulo, Brazil; ^4^ Laboratory of Neuroscience and Behavior; University of Brasilia, Brasilia, Brazil; ^5^ Biobank for Aging Studies of the University of São Paulo, São Paulo, São Paulo, Brazil; ^6^ Faculty of Medicine, Euro‐American University Center‐UNIEURO, Brasilia, Brazil

## Abstract

**Background:**

Cerebral amyloid angiopathy (CAA) is characterized by accumulation of β‐amyloid (βA) protein in cerebral vessels. Animals models are important for studies in AD and CAA. However, among the animal models that have been described, none developed CAA‐related intracerebral hemorrhage. We analyze the brain of a 29‐year‐old female capuchin monkey who spontaneously developed CAA‐associated vascular changes.

**Method:**

After brain extraction, the whole brain was fixed in 4% buffered paraformaldehyde within 12 hours of death. 7T MRI were acquired before and after brain slicing. Consecutive coronal sections from the fixed brain were embedded in paraffin, and 5µm sections from paraffin blocks were used for staining and immunohistochemistry evaluation. All brain sections were immunostained with hematoxylin & eosin, and with antibodies against the following proteins: βA protein (βA; 4G8), phospho tau (AT8) and ubiquitin‐proteosome (p62). Also, additional immunostaining analysis using TDP‐43, α‐synuclein (81A), GFAP, Iba‐1, βA40 and βA42 antibodies, and Masson Trichrome and Perls stains were performed in selected areas.

**Result:**

AD‐type pathology equivalent to Braak V, CERAD B and Thal phase 3 (A2B3C2) was observed. Additionally, βA deposits were noted in meningeal and parenchymal arterioles as well in numerous parenchymal capillaries of several areas of the brain, predominantly in the neocortex. Other areas that developed CAA include the cerebellum, hippocampus, amygdala, basal ganglia, diencephalon and, very rarely, the brainstem. Parenchymal and meningeal vessels were immunoreactive for βA40 and βA42. Whereas both isoforms were found in arterioles and capillaries, there was a predominance of βA40 compared to βA42. 7T MRI demonstrated an old hematoma, characterized as low foci in SWI and T2 images with central cavitation, alongside multiple smaller areas of old hemorrhage. Hematoxylin & eosin staining revealed cystic microcavitation with astrocytic gliosis and hemosiderin deposit. Besides, microbleeds with hemosiderin deposits were found in cerebellum and concentric arteriolosclerosis was observed mainly in basal ganglia and thalamus.

**Conclusion:**

AD and CAA pathological changes similar to those observed in human brains have been observed. Furthermore, the presence of neuroinflammation markers and βA40 and 42 isoforms suggest that this New World monkey could be a model not only for AD, but also for CAA studies.

